# Rapid Quantitative Analysis of IR Absorption Spectra for Trace Gas Detection by Artificial Neural Networks Trained with Synthetic Data

**DOI:** 10.3390/s22030857

**Published:** 2022-01-23

**Authors:** Jens Goldschmidt, Leonard Nitzsche, Sebastian Wolf, Armin Lambrecht, Jürgen Wöllenstein

**Affiliations:** 1 Laboratory for Gas Sensors, Department of Microsystems Engineering-IMTEK, University of Freiburg, Georges-Köhler-Allee 102, 79110 Freiburg, Germany; juergen.woellenstein@ipm.fraunhofer.de; 2 Fraunhofer Institute for Physical Measurement Techniques IPM, Georges-Köhler-Allee 301, 79110 Freiburg, Germany; leonard.nitzsche@ipm.fraunhofer.de (L.N.); sebastian.wolf@ipm.fraunhofer.de (S.W.); armin.lambrecht@ipm.fraunhofer.de (A.L.)

**Keywords:** spectral analysis, quantitative gas analysis, machine learning, artificial neural networks, dual comb spectroscopy, broadband spectroscopy, laser spectroscopy

## Abstract

Infrared absorption spectroscopy is a widely used tool to quantify and monitor compositions of gases. The concentration information is often retrieved by fitting absorption profiles to the acquired spectra, utilizing spectroscopic databases. In complex gas matrices an expanded parameter space leads to long computation times of the fitting routines due to the increased number of spectral features that need to be computed for each iteration during the fit. This hinders the capability of real-time analysis of the gas matrix. Here, an artificial neural network (ANN) is employed for rapid prediction of gas concentrations in complex infrared absorption spectra composed of mixtures of CO and N_2_O. Experimental data is acquired with a mid-infrared dual frequency comb spectrometer. To circumvent the experimental collection of huge amounts of training data, the network is trained on synthetically generated spectra. The spectra are based on simulated absorption profiles making use of the HITRAN database. In addition, the spectrometer’s influence on the measured spectra is characterized and included in the synthetic training data generation. The ANN was tested on measured spectra and compared to a non-linear least squares fitting algorithm. An average evaluation time of 303 µs for a single measured spectrum was achieved. Coefficients of determination were 0.99997 for the predictions of N_2_O concentrations and 0.99987 for the predictions of CO concentrations, with uncertainties on the predicted concentrations between 0.04 and 0.18 ppm for 0 to 100 ppm N_2_O and between 0.05 and 0.18 ppm for 0 to 60 ppm CO.

## 1. Introduction

Quantitative gas analysis for the determination of the concentration of gases present in the sample has emerged as a powerful tool in a variety of applications in environmental sensing [1,2,3], medicine [4,5], or process monitoring [6]. Well-established techniques such as mass spectrometry and gas chromatography offer high sensitivity and selectivity even in highly complex gas matrices, but do not support high acquisition rates [7,8,9,10]. By comparison, optical techniques based on infrared (IR) absorption spectroscopy offer fast measurement times in addition to high sensitivity. They make use of the absorption of light in the sample at wavelengths that are characteristic for the illuminated gas species [11]. One issue in the use of optical techniques is that complicated gas matrices with more than one infrared-active component can lead to overlapping absorptions and hence to increased cross-sensitivities [12]. To circumvent this, it is useful to increase the spectral bandwidth to be able to reliably distinguish the different gas species. To determine the concentrations of the gases of interest, fitting algorithms based on non-linear least squares are often employed [13,14], relying on absorption profiles provided by spectroscopic databases such as HITRAN [15]. The extended spectral bandwidth, however, leads to an increased number of absorption profiles, which have to be computed for every iteration during a fit. This drastically increases the computation time to retrieve the concentration information. Furthermore, broadband spectra also suffer from complex background structures. These need to be corrected in pre-processing or also considered by the fit hypothesis, which increases the number of free parameters, leading to more required iterations during fitting and hence to an increased computation time. This makes it a difficult task to reliably detect and fit weakly absorbing gases in the spectrum, posing an obstacle to rapid quantitative gas analysis, which is needed especially for applications such as closed-loop process control and for monitoring of dynamic processes.

With the use of machine learning-based methods, it is possible to build regression models, e.g., partial least-square regression (PLSR), for the prediction of gas concentrations [16]. In particular, models based on artificial neural networks (ANNs) have shown promising results in building regression models and processing acquired data in real time [17,18]. ANNs consist of nodes that are typically arranged in layers. During training the strengths of the connections (weights) between the different nodes and layers are adjusted to minimize the deviation of the ANNs predictions to the target values, which are attached as so-called labels to the training data. Training of these network structures typically requires large datasets (often in the order of hundreds of thousands of data, or more) with a high variety of concentrations and mixtures, setting a high experimental burden for the preparation of actual measurements. Data augmentation techniques, for example, can be used to increase the amount of training data by linear combination of measured spectra [19,20].

In this work, we present a different approach for rapid quantitative analysis of molecular absorption spectra based on a feed-forward artificial neural network building a regression model. The network is trained purely with synthetic infrared absorption spectra instead of experimentally recorded datasets, following a similar approach described by Kern et al., where they utilized synthetic training data based on spectral models for NMR spectra [21]. In our approach, the synthetic spectra are composed of absorption features described by Voigt profiles calculated with the HITRAN API [22]. In addition, we also take into account the dual comb spectrometer’s properties, e.g., resolution or noise equivalent absorption, and additional influences on the spectra, e.g., optical fringes. The synthetic training data are generated in the Fourier-transformed domain, as opposed to recorded time domain interferograms (as demonstrated by Voumard et al. [18]). Thus, only the instrument influences and parasitic signals within the observed spectral band have to be considered, and other contributions to the interferograms, such as radio station signals or parasitic signals from surrounding electronics, which couple into the signal lines, can be neglected.

The proposed synthetic training was evaluated on measurements taken with a mid-infrared dual frequency comb spectrometer, suited for the analysis of multi-component gas mixtures [23,24]. For the application demonstration, the spectrometer system was set up for the detection of CO and N_2_O in a wavelength band around 4.57 µm, which shows overlapping absorption profiles for both gases. First, the spectrometer’s properties and influence on the measured spectra were investigated. A quantity of 10^7^ synthetic spectra with arbitrary concentrations between 0 to 60 ppm and 0 to 100 ppm for CO and N_2_O, respectively, were generated and superimposed by a baseline with arbitrarily chosen parameters in a range, which was known from the previous investigation of the spectrometer. This approach is only valid for trace gas concentrations, as higher concentrations would lead to distortions of absorption profiles, e.g., due to changes of broadening coefficients. The network was trained and validated with generated synthetic spectra and tested using spectra measured with the dual comb spectrometer. We then compared the ANN’s predictions on the measured spectra with the results given by a classical fitting algorithm in terms of uncertainty, linearity, and calculation time. There already exist several dedicated systems and approaches for the precise determination of N_2_O and CO [25,26]. This use case, however, exemplifies the potential of the proposed method for the analysis of absorption spectra within a specific spectral band.

## 2. Methods

### 2.1. Experimental Setup

**Figure 1:**
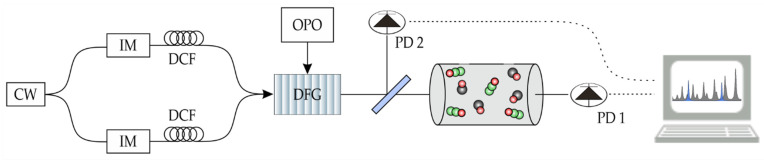
Schematic setup of the spectrometer. The light emitted by a continuous wave laser (CW) is split into two branches and modulated by two intensity modulators (IM) with slightly different repetition rates. The resulting combs are spectrally broadened in dispersion-compensating fibers (DCF), superimposed and converted to the mid-infrared via difference frequency generation (DFG) with an optical-parametric oscillator (OPO) in a periodically poled lithium niobate crystal. The mid-infrared dual comb is divided by a beam splitter and detected with PD 2 before the gas cell and after with PD 1.

Experimental data for quantitative evaluation and testing of the ANN were obtained from a mid-infrared dual frequency comb spectrometer. The spectrometer is based on electro-optic modulation of a common continuous wave laser at 1550 nm, subsequent spectral broadening in dispersion-compensating fibers, and conversion into the mid-infrared by difference frequency generation with a tunable pump source. A detailed description of the spectrometer can be found in [23]. The central wavelength of the spectrometer is set to 4.57 µm with a spectral coverage of 150 GHz (5 cm^−1^) and a mode spacing of 500 MHz (0.017 cm^−1^), which defines the effective frequency resolution. The mode spacings of the single combs are slightly different—in the order of kHz—which results in a beat signal between both combs and hence in a train of interferograms [27]. In the mid-infrared, the dual comb is split into two branches. One branch is detected by a HgCdTe detector (PVI-4TE/VIGO Systems) as a reference. The other branch is passed through a multi-reflection cell with an absorption length of 720 cm and detected with a second detector behind the cell (see Figure 1). The cell is filled with mixtures of CO and N_2_O. The concentrations are set using three mass flow controllers (EL Flow Prestige/Bronkhorst), which mix two premixtures of 60 ppm CO in synthetic air and 100 ppm N_2_O in nitrogen. For lower target gas concentrations, these mixtures are further diluted by adding pure nitrogen. Due to the mutual coherence of the combs [24], coherent averaging can be employed before computing the power spectra for each of the individual channels via fast-Fourier-transformation (FFT). From the spectra, only the maximum positions of the comb modes are selected, which yields the respective sample $S_{\mathrm{sample}}$ and reference $S_{\mathrm{ref}}$ spectra. The coherent averaging and comb mode selection allow compressing the recorded data stream from 1 GByte/s down to 10 kByte/s, enabling the continuous spectra acquisition with a 10 Hz rate [28]. Absorbance spectra are obtained by normalization of the sample spectrum derived from the recorded signal of PD 1 by the spectrum derived from the signal of PD 2 given with [12]:

(1) $$A=-\ln\left(\frac{S_{\mathrm{sample}}}{S_{\mathrm{ref}}}\right).$$

**Figure 2:**
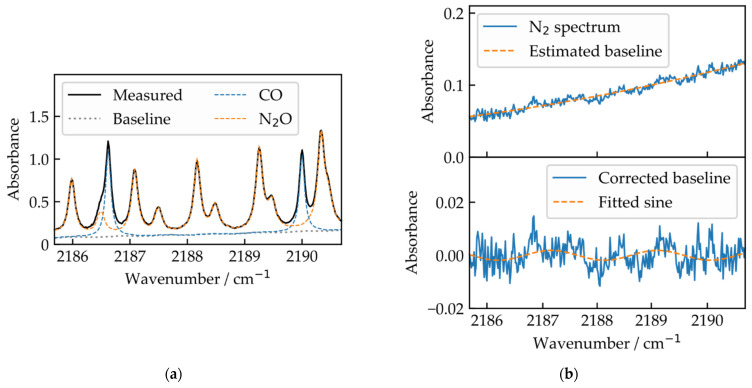
(**a**) measured absorbance spectrum of a gas sample with target gas concentrations of 30 ppm for CO and 50 ppm for N_2_O. The spectrum is recorded with a dual comb spectrometer comprising 301 spectral elements separated by 0.017 cm^−1^; (**b**) exemplary measured nitrogen spectrum, which is not infrared-active. From these, the spectrometer influence on the measured absorbance spectrum is estimated by fitting and subsequent normalization using a polynomial function (top) and additional sine functions (bottom) to model optical fringes.

Figure 2a shows an exemplary measured absorbance spectrum for 50 ppm N_2_O and 30 ppm CO composing 301 spectral elements.

### 2.2. Description of the Fitting Algorithm and Raw Data Analysis

Classical fitting algorithms are based on optimizing a parameter set β for a given model function f(x,β), describing the underlying data with N datapoints $\left(x_{N},y_{N}\right)$. This is usually performed by using non-linear least squares, where the residual sum of squares $SS_{\mathrm{res}}=\overset{N}{\underset{i}{\sum }}{\left(y_{i}-f\left(x_{i},\beta \right)\right)}^{2}$ is minimized. For this, it is beneficial that the initial parameters for the fitting procedure are already a coarse approximation of the fit results. Otherwise, the fit may not converge or a local minimum in $SS_{\mathrm{res}}$ may stop the algorithm, resulting in incorrect or unreasonable fit results.

To validate our approach using ANNs trained with synthetic data to evaluate infrared absorption spectra, we compared it to a non-linear least squares fitting algorithm implemented in the Python library SciPy [29]. The model function to fit the measured spectra is therefore given with:

(2) $$f_{\mathrm{fit}}\left(\nu ,\nu_{0},c_{\mathrm{CO}},c_{N2O},\;\vec{b}\right)=\alpha \left(\nu -\nu_{0},c_{\mathrm{CO}},c_{N2O}\right)L+baseline\left(\nu ,\;\vec{b}\right).$$

This function includes the gas absorption profiles α based on HITRAN [15], gas concentrations $c_{\mathrm{CO}}$ and $c_{N2O}$, the absorption path length L, which is kept constant at 720 cm, and a global wavenumber shift $\nu_{0}$. To increase robustness against additional absorbers, e.g., water, the theoretical model (Equation (2)) can be expanded. Additionally, the normalization of the spectra (given by Equation (1)) is imperfect due to a power mismatch on the detectors in addition to optical fringes, e.g., caused by the lithium niobate crystal. We treat these effects as additional baseline contributions, which must be included in the fit model. The baseline contribution is demonstrated on a measured absorbance spectrum for 30 ppm CO and 50 ppm N_2_O, depicted in Figure 2a. The baseline function,

(3) $$baseline\left(\nu ,\vec{b}\right)=b_{0}+b_{1}\nu +b_{2}\nu^{2}+b_{3}\sin\left(b_{4}\nu +b_{5}\right)+b_{6}\sin\left(b_{7}\nu +b_{8}\right)$$

consists of a polynomial of second order—several orders were tested, this gave the best results according to RMS-noise in the residuals—and two sine functions, where the amplitudes, phases, and frequencies are considered, to account for the apparent optical fringes in the spectrum, resulting in nine free baseline parameters ($\vec{b}$).

To be able to retrieve the information of the spectrometer’s influence on the spectra isolated from gas absorptions, the described fitting procedure was applied to pure nitrogen spectra, where no absorbing gas is present. This allowed us to quantify the variations of the parameters, to be able to consider this in the synthetic training data for the ANN. For this, we measured 100 nitrogen spectra, where an exemplary spectrum is shown in Figure 2b, and fitted them with Equation (3), as no absorbing gases are present and therefore no concentrations or wavenumber shifts can be fitted. The information on the range of the global wavenumber shift in the spectra is determined by evaluating slow wavelength drifts of the OPO and the seed-laser of the near-infrared dual comb with long term wavelength measurements. The limits of the variations of the baseline parameters, and the limits for the wavenumber shifts, are shown in Table 1. These limits are expanded by 20% to increase robustness of the ANN against variations potentially undetected by the fit. The parameter analysis for this dual comb setup tuned to this particular spectral region limits the parameter space as the variations of the respective parameters are limited. We assume that expanding the parameter space is possible, but would require a longer training or a larger set of training data, and was not undertaken for this demonstration.

### 2.3. Artificial Neural Network Description

**Figure 3:**
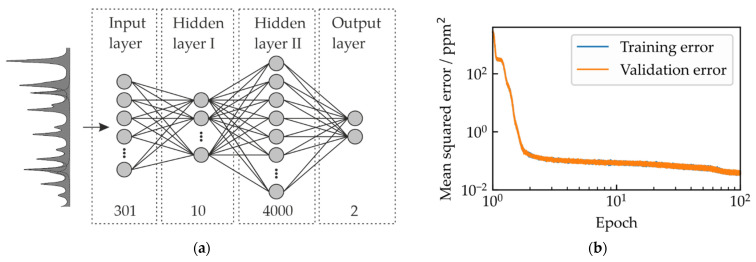
Artificial neural network architecture and training results. (**a**) The spectrum with 301 spectral points is put into the first network layer, whose number of nodes corresponds to the number of spectral points. The network consists of two hidden layers with 10 and 4000 nodes for the first and second layer, respectively. The network is trained to provide the gas concentrations in units of parts per million (ppm) in the output layer; (**b**) Evolution of the mean squared error while network training for the training and the validation dataset. The number of epochs is defined by the number of iterations on the entire training dataset, which contains 10^7^ labeled synthetically generated spectra.

The ANN architecture is set up as a fully connected, feed-forward neural network with two hidden layers (Figure 3a) making use of the Python library PyTorch [30]. The input layer consists of 301 nodes, which correspond to the number of spectral elements (comb modes) in the measured spectra. A range of configurations of nodes in the hidden layers were tested for this specific case. Here, 10 nodes in the first and 4000 in the second hidden layer resulted in a minimum achievable deviation of the predicted concentrations from the target concentrations. The output layer consisting of two nodes directly yields the information of interest, namely the concentrations of CO and N_2_O. For the activation function we chose the rectified linear unit. For optimization during training the back propagation algorithm Adam [31] is used and the loss function is defined by the mean squared error loss (MSE) given by:

(4) $$MSE=\frac{1}{N}\sum_{i}^{N}{\left(y_{i}-{\hat{y}}_{i}\right)}^{2},$$

with the number of predictions N, the predicted concentration $y_{i}$ and the target concentration ${\hat{y}}_{i}$.

### 2.4. Synthetic Data Generation and Network Training

To train the ANN, we first simulate two isolated absorbance spectra with 301 spectral points (150 GHz/5 cm^−1^ spectral coverage), an absorption path length of 720 cm, and gas concentrations of 1 ppm for both CO and N_2_O. To account for the random global wavenumber shift $\nu_{0}$, the synthetic spectra are shifted pairwise by 10 MHz (0.00033 cm^−1^). To cover the wavenumber range determined before, this results in 600 spectra pairs, where, in each pair, CO and N_2_O are shifted by the same amount. The temperature and pressure of the spectra are set to 299.3 K and 0.982 atm, which we measured in the gas cell while acquiring the test spectra for this proof-of-principle. However, when samples with different pressures and temperatures are investigated, those parameters would also have to be varied. We then set 10^7^ labels for both gases ranging from 0 to 110 for N_2_O and 0 to 66 for CO respectively. These labels were also used to rescale the basic absorbance spectra for 1 ppm to the respective concentration. Within these ranges, the labels are generated randomly following a uniform distribution. This ensures that each subset of the respective concentrations range is prioritized equally. Again, the limits are expanded by 10% to train the ANN more robust against unexpected variations. We do not expect larger deviations from the setpoints given by the error budget, comprising an uncertainty of 2% on the concentrations of the premixtures, and of 0.5% on the MFC flows (according to the manufacturer), an uncertainty of 2% on the absorption path length, and additional uncertainties on the line intensities (2 to 5% for N_2_O and <1% for CO [15]), where uncertainties on the measured pressure and temperature are negligible. In each training step, a randomly shifted spectrum pair is picked and multiplied by a random label for each gas, as the absorbance scales strictly linear with the gas concentration, and both spectra are combined. The resulting mixture spectrum is then superimposed with a random baseline, whose parameters are randomly chosen with a uniform distribution between the values given in Table 2, to construct a synthetic spectrum, which is inserted into the network after adding noise. The synthetic noise follows a normal distribution with a sigma value of 1.45 × 10^−2^ which is derived from the noise equivalent absorbance of the spectrometer for 0.1 s [23]. At each 10th iteration within an epoch, where an epoch is defined by an iteration over 10^7^ datasets, the training is stopped and the model is evaluated with validation data, that are synthesized the same way as the training data but are never seen by the model while training. This is ensured by generating the validation data independently from the training data and by randomly picking and compiling new sets of spectra at each validation step. The network is trained over 100 epochs on a NVIDIA Quadro P4000 graphics unit within 36 h of training time. The evolving mean squared error (MSE) while training is depicted in Figure 3b, where the MSE is a measure for the performance of the ANN on the training and validation data. Although the MSE only decreases moderately after the third epoch, it was chosen to train the ANN within 100 epochs, to check if the MSE starts to oscillate or overfitting of the regression model occurs, where the ANN is forced to learn too many details of the training data, leading to a decreased performance in generalization and hence to a divergence of the training and the validation MSE. In our case, neither oscillations of the MSE nor overfitting are observed, even up to 500 epochs of training.

## 3. Results and Discussion

### 3.1. Network Evaluation on Measured Spectra

To study the performance of the ANN, namely uncertainties of the determined concentrations, linearity, and computation time, and to be able to compare it to the results obtained by a fit with Equation (2), we measured absorbance spectra of 18 different mixtures of N_2_O and CO with concentrations between 0 and 100 ppm for N_2_O and 0 and 60 ppm for CO, with a recording time per mixture of 10 s (100 acquired spectra per mixture). The different mixtures are listed in Table 2, where all set flows of the MFCs are within the specified operational range (20 to 1000 mL/min) provided by the manufacturer.

**Figure 4:**
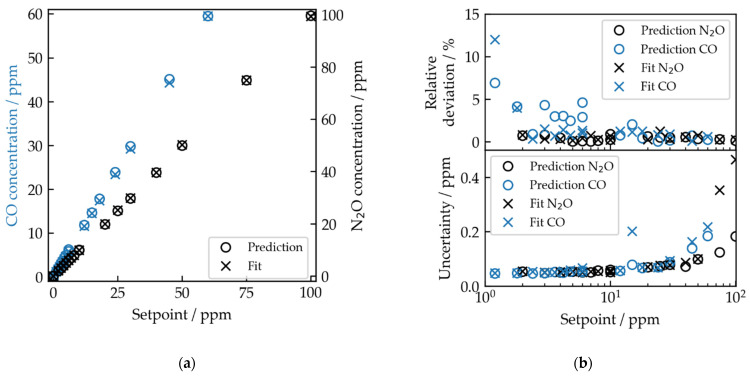
(**a**) Results for the fitted (crosses) and predicted (circles) concentrations for CO and N_2_O. The determined concentrations are corrected by a factor, derived from the difference of the concentrations of the pure premixtures and the expected value; (**b**) relative deviation of the determined concentrations to the linear fits and uncertainty for the ANN predictions and fit results, given by the standard deviation of the calibrated concentrations for each mixture.

The concentrations resulting from the fit (crosses) and predicted by the ANN (circles) are depicted in Figure 4a. The determined concentrations are corrected by a factor, which is derived from the difference between the expected and the concentrations determined by the fit and the ANN of the pure sample gases, as the expected concentrations are not known accurately due to the error budget described above. We perform a linear fit with the equation

(5) $$f_{\mathrm{lin}}\left(c_{\mathrm{set}}\right)=mc_{\mathrm{set}}+y_{\mathrm{int}},$$

where $c_{\mathrm{set}}$ represents the concentration setpoints, m is the slope, and $y_{\mathrm{int}}$ is the *y*-axis intercept. With this we determine the coefficient of determination given by

(6) $$R^{2}=1-\frac{SS_{\mathrm{res}}}{SS_{\mathrm{tot}}}$$

with the residual sum of squares $SS_{\mathrm{res}}=\overset{N}{\underset{i}{\sum }}{\left(y_{i}-{\hat{y}}_{i}\right)}^{2}$ and the total sum of squares $SS_{\mathrm{tot}}=\overset{N}{\underset{i}{\sum }}{\left(y_{i}-\bar{y}\right)}^{2}$, where N is the number of samples, $y_{i}$ the predicted (fitted) concentration given by the ANN (fit), ${\hat{y}}_{i}$ the estimated concentration given by the linear fit, and $\bar{y}$ the global mean value of the predicted (fitted) concentrations. The resulting coefficients of determination are listed in Table 3. Figure 4b shows the relative deviation of the calibrated concentrations $c_{\mathrm{fit}}$ and $c_{\mathrm{ANN}}$ from the linear fit—which provides information for the linear behavior of the predictions and fit results—in addition to the uncertainties on $c_{\mathrm{fit}}$ and $c_{\mathrm{ANN}}$. The uncertainties are calculated by the standard deviation of $c_{\mathrm{fit}}$ and $c_{\mathrm{ANN}}$.

The relative deviations decrease with increasing gas concentrations. The minimum relative deviations from the linear fit, in addition to the minimum and maximum observed uncertainties, are listed in Table 3. The uncertainties of the fit results and ANN predictions stay constant around 0.05 ppm until 10 ppm. For higher concentrations, the uncertainty increases to more than two times the value for N_2_O for the fit results. Additionally, an unexpected increase at 15 ppm of the fit uncertainty is visible.

Between 3 and 6 ppm, an increase in the relative deviation of CO predictions from the linear fit is observed. This may result from the random selection of synthetic training spectra, as the network was trained with too few spectra in this concentration range, and hence is less robust against baseline contributions. The stronger impact on the CO predictions compared to the N_2_O predictions may stem from the fact that the area under the CO absorptions—where the area under the absorption is proportional to the respective gas concentration—is smaller compared to the area under N_2_O absorptions (see Figure 2a), due to fewer absorption lines of CO in this spectral region. This leads to an increased influence of the baseline to the network prediction. Additionally, the CO line around 2187.5 cm^−1^ strongly overlaps with a weaker N_2_O line, which increases the cross-sensitivity of the CO predictions for lower CO concentrations. This will be investigated further in the future with different sets of test spectra, e.g., CO and CO_2_ or N_2_O and CO_2_.

The ANN predictions show a linear behavior with uncertainties comparable to the fit for gas concentrations up to 10 ppm. For higher gas concentrations, the uncertainties of the ANN predictions are lower compared to the fit, indicating a higher repeatability of the results. However, the ANN is susceptible to systematic deviations due to the training approach with randomized synthetic data. The achieved levels of relative uncertainty on the predictions compare well to the results of a PLSR-based evaluation of CO and N_2_O concentrations from quartz-enhanced photoacoustic spectroscopy (QEPAS) measurements in the same spectral region [16]. In contrast, the absolute uncertainties of the ANN approach presented here consistently lie in the ppb range and also demonstrate applicability for lower concentration ranges. An in-depth comparison of the methods, however, would require additional considerations of the underlying instrumentation.

The computation time per spectrum is reduced from 484 s on average for the used fit to 303 µs on average for the ANN predictions. This enables real-time evaluation of acquired spectra with a maximum rate of 3 kHz without further optimization or dedicated hardware, e.g., FPGAs. An additional advantage of the proposed method is that classical fitting algorithms need prior suggestions on the parameters, which have to be fitted. Unsupervised problems, where no coarse prior knowledge on the concentrations in the sample is available, can lead to an increased computation time, unreasonable fit results, or a failure of the fit.

It has to be mentioned that evaluation times of the fit can be decreased using more dedicated hardware or by further optimization of the fitting algorithm. For example, Tao et al. showed computation times of comparable spectra of a mixture of N_2_O and CO down to 0.1 s with a dedicated numerical fitting model for wavelength modulation spectroscopy [26]. Nevertheless, the fit used in our work enabled a true comparison of the ANN and fitting approach in a closed system as the synthetic training data are based on the same model as the fit function.

### 3.2. Unsupervised Evaluation of a Dynamic Process

To test the performance of our implemented method on an unsupervised dynamic process, we evaluated the gas exchange in the cell when switching from pure nitrogen to a preset gas mixture. The gas cell was therefore completely purged with nitrogen so that no absorbing gas is left in the cell. We then filled the cell with a flow rate of 500 mL/min for each gas (total flow rate of 1 L/min) and recorded spectra with a 10 Hz rate with 100 s of acquisition, where the flow was started roughly 7 s after data acquisition. The gas concentrations during gas exchange were unknown, except for the start and end point of data acquisition.

**Figure 5:**
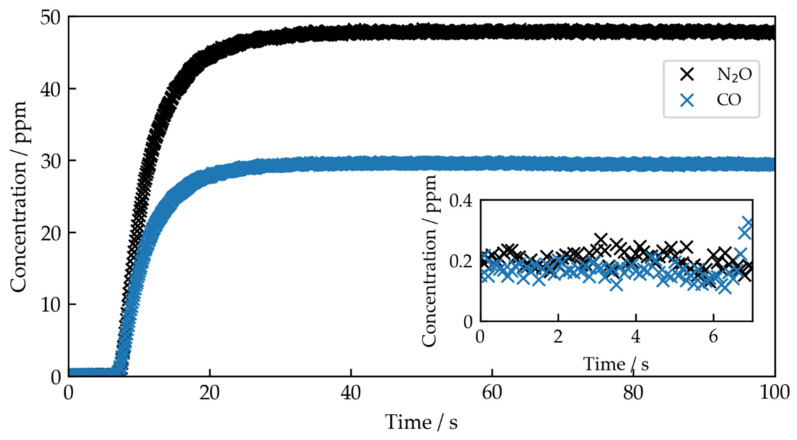
ANN predictions of gas concentrations during gas exchange of the gas cell, which was initially filled with nitrogen and then flushed with a mixture of 30 ppm CO and 50 ppm N_2_O. The inset shows the first 7 s of the measurement, where only nitrogen is present in the cell.

The ANN was trained again as described previously on 10^7^ synthetic spectra. The variations of the gas concentrations were adapted to new concentration ranges up to a maximum of 55 ppm for N_2_O and 33 ppm for CO. The acquired spectra were then evaluated by the ANN. The resulting predictions for the gas concentrations are shown in Figure 5. To identify the time to reach the target concentrations of 50 ppm N_2_O and 30 ppm CO we fit the function

(7) $$f\left(t,t_{0},c_{\mathrm{gas}},\tau \right)=c_{\mathrm{gas}}(1-\exp\left(-\left(t-t_{0}\right)k\right)$$

to both curves, with time t, the time where the gas exchange started $t_{0}$, the exchange rate k, and the target gas concentration $c_{\mathrm{gas}}$. The resulting fitted target concentrations (fit uncertainties) are 47.585(2) ppm and 29.428(1) ppm with the times to reach 99% of the target gas concentrations of 20.3 s and 19.3 s for N_2_O and CO, respectively. The average predicted concentrations after 20.3 s are 47.66 ppm for N_2_O and after 19.3 s 29.46 ppm for CO. We observe a relative deviation of the determined target gas concentrations by the ANN predictions compared to the expected values of 4.68% for N_2_O and 1.80% for CO. The deviations from the expected values lie within the expected systematic error, which can be estimated as 6% on the set N_2_O concentration and 4% on the set CO concentration, resulting from the previously described error budget. These results are comparable to the deviations of the predicted concentrations of the pure premixtures, which have found to be 4.27% for N_2_O and 1.47% for CO.

The inset in Figure 5 shows the predicted concentrations in the first 7 s, where only nitrogen should be present in the cell. However, the ANN predicts concentrations around 0.21 ppm for N_2_O and 0.17 ppm for CO. To investigate this, we gradually decreased the number of synthetic spectra for training in steps of 10^5^ from 10^7^ to 10^5^. For instance, we observe an increase in the offset to 0.46 ppm for N_2_O and 0.24 ppm for CO with 10^6^ spectra and to 1.11 ppm for N_2_O and 0.75 ppm for CO with 10^5^ spectra. This indicates that the offset depends on the quantity of training data, converging towards zero for larger datasets. How this effect can be further minimized will be part of future investigations, e.g., by using dedicated data augmentation techniques, as presented in [32].

The observed offset influences the limit of detection (LOD) of the method. For comparison, the LOD (3σ) of the spectrometer for spectra acquired with a 10 Hz rate was found to be 0.36 ppm for N_2_O and 0.14 ppm for CO, which was calculated using the standard deviation (σ) of the determined concentration, where no absorbing gas was present [33]. Following the same approach to calculate the LOD for the ANN predictions, we found 0.09 ppm for N_2_O and 0.06 ppm for CO, which results in 0.30 and 0.23 ppm, respectively, when we also take into account the observed offsets. Thus, the use of the ANN does not increase the LOD of the system, but introduces a systematic offset, which has to be considered.

## 4. Conclusions

We present a method for rapid analysis of broadband mid-infrared absorption spectra for trace gas detection enabled by a feed-forward artificial neural network (ANN). The collection of huge amounts of training data under variation of target gas concentrations is avoided by utilizing synthetic mid-infrared spectra with absorption profiles given by HITRAN. For training of the network, these synthetic spectra are augmented by allowing for shifts in their spectral position, considering baseline effects with a polynomial function and approximating optical fringes with sine functions. This reproduces the underlying impact of the used spectrometer—namely a mid-infrared dual comb spectrometer—on measured spectra. The variation ranges of these effects were approximated a priori by classical fitting methods on nitrogen spectra, which led to a drastically decreased set of calibration spectra, as no concentration variations had to be covered. The implemented artificial neural network builds a regression model and consists of two hidden layers. The network is trained within 36 h and tested with measured infrared absorption spectra of mixtures of gases CO and N_2_O with concentrations from 0 to 60 ppm and 0 to 100 ppm, respectively. The network predictions show linear behavior with coefficients of determination of $R^{2}{}_{N_{2}O}=0.99997$ and $R^{2}{}_{\mathrm{CO}}=0.99987$, and uncertainties on the predictions ranging from 0.05 to 0.18 ppm for concentrations from 0 to 60 ppm CO, and uncertainties on the predictions from 0.04 to 0.18 ppm for concentrations ranging from 0 to 100 ppm N_2_O. These results are comparable to a classical evaluation based on a non-linear least squares fit. With the ANN, the computation time to analyze a single spectrum is decreased from 484 s on average per spectrum to 303 µs per spectrum on average. The high computation times of the classical evaluation result from the absence of reference spectra (where no absorbing gas is present), as complex baseline contributions to the spectra had to be covered in the fit, whereas the ANN is trained robustly against these contributions. This renders the recurrent gas exchange of the sample gases with nitrogen as in [23,33] unnecessary, hence reducing the experimental effort needed to acquire spectra. Additionally, no initial approximations on the gas concentrations are needed to reliably evaluate the spectra. The method competes well against multivariate analysis in terms of prediction uncertainty. However, the synthetic data-based training approach here further reduces the reliance of training on experimental data compared to the existing literature. The spectra analysis rate is currently limited by the spectra acquisition rate of the dual comb spectrometer and the algorithm for continuous data acquisition. Nonetheless, we assume that the proposed approach can also be applied to other spectrometer types such as tunable laser absorption spectrometers, allowing higher acquisition rates. This shows the potential of ANN-based methods as a promising instrument towards rapid and precise analysis of gases and gas mixtures.

## Figures and Tables

**Table 1:** Determined limits of the parameter space for the generation of the training data, baseline parameters and wavenumber shift ranges.

	Minimum Value	Maximum Value
Wavenumber shift (cm^−1^)	−0.1	0.1
*b*_0_	$6.041\times 10^{-2}$	$2.012\times 10^{-1}$
*b*_1_ (cm)	$1.694\times 10^{-4}$	$8.201\times 10^{-4}$
*b*_2_ (cm^2^)	$-3.533\times 10^{-7}$	$8.870\times 10^{-7}$
*b*_3_	$3.915\times 10^{-4}$	$8.387\times 10^{-3}$
*b*_4_ (cm)	−1.601	$6.662\times 10^{2}$
*b*_5_	$-1.428\times 10^{2}$	$2.644\times 10^{2}$
*b*_6_	$5.111\times 10^{-4}$	$7.89\times 10^{-3}$
*b*_7_ (cm)	$3.149\times 10^{-1}$	$6.229\times 10^{-1}$
*b*_8_	$-1.444\times 10^{2}$	$2.132\times 10^{2}$

**Table 2:** Setpoints for measured mixtures of N_2_O and CO.

Mixture	1	2	3	4	5	6	7	8	9	10	11	12	13	14	15	16	17	18
Set N_2_O concentration (ppm)	0	100	50	25	75	10	40	20	30	0	10	2	8	3	7	4	6	5
Set CO concentration (ppm)	60	0	30	45	15	24	6	18	12	6	0	4.8	1.2	4.2	1.8	3.6	2.4	3

**Table 3:** Coefficients of determination, minimum achieved relative deviations, and minimum and maximum uncertainties resulting from ANN predictions and fits.

	ANN		Fit	
	N_2_O	CO	N_2_O	CO
$R^{2}$	0.99997	0.99987	0.99973	0.99991
Minimum relative deviation (%)	0.03	0.04	0.14	0.09
Minimum/maximum uncertainty (ppm)	0.04/0.18	0.05/0.18	0.01/0.47	0.03/0.22

## Data Availability

Data and code underlying the presented results are not available publicly. They may be delivered by the corresponding author upon reasonable request.
